# On the Physiological Action of the Muscles Which Control and Influence the Lower Jaw

**Published:** 1868-05

**Authors:** Thomas Brian Gunning


### ARTICLE II.

### *On the Physiological Action of the Muscles which control and influence the Lower Jaw.*

BY THOMAS BRIAN GUNNING.

(Concluded.)

THE manner in which the head is controlled will be better understood, by giving some attention to the action of these and other muscles of this region, in rising from lying on the back, and when the head is brought forward from its most backward position, with the body upright. In rising from the back, the sterno mastoid-muscles bring up the atlas, by means of the mastoid processes, but the weight of the head, beyond the atlas, requires a counterpoise; this is found in the genio-hyoid, which comes into action and assists until the body is upright, and the head in balance; in doing this, its action is direct between the jaw, and the vertex of the angle formed in the hyoid bone by the omo-hyoid, and the posterior belly of the digastric muscles. It is probable that the superior constrictors, which pass between the back of the pharynx and lower jaw, etc., and the middle constrictors, which are attached to the hyoid bone, also assist.

With the body upright and the head over back, however, the chin is thrown up, and the genio-hyoid has approached the line of the sterno-hyoid, which last assists in fixing the hyoid bone, which has now considerable strain upon it in a vertical direction. This strain can be safely met as the sterno-hyoid muscle is inserted into the front of the body of the hyoid bone immediately under the genio-hyoid. As the head comes forward into balance, it is surrendered to the digastric and omo-hyoid muscles. The relaxation of the genio-hyoid muscle, can be distinctly felt with the finger; and singing the higher notes of the voice, will afford comparison between its full action and its inactivity or indifference to the head, after the latter is upright, even with the chin drawn in forcibly; whereas, in this same position, the omo-hyoid and digastric are very strongly in action, a fact which is proved by applying the tongue against the inside of the upper front teeth, and exerting strong suction repeatedly while the fingers are placed upon the muscles as before directed. It will then be found that little movement is made by either muscle, compared with what is felt, when the chin is in its proper position; thus showing that these muscles are so strongly engaged, as to be unable to increase their action much in sucking, although this function calls them into action more strongly than any other, when the head is naturally placed, and, as it is their co-operation can be readily distinguished. Further, the omo-hyoid can be distinctly felt rising above the clavicle, in sitting down or getting up, with the head in the natural position; whereas, with the head thrown back the omo-hyoid is so low that it can scarcely be felt, it being with the digastric relaxed somewhat, to allow the head to go back. The sterno-thyroid muscle gives little or no assistance during these movements of the head and body, it can however, be felt decidedly in singing the higher notes or in sucking and more distinctly in deglutition, by placing the finger in the inter-clavicular notch. The thyro-hyoid muscle assists but little, if any, in these movements of the head and body, and in that of sucking; but in singing, and especially in deglutition, its action is very strong. This can be felt by placing the finger on the thyro-hyoid membrane.

In all positions of the head, the lower jaw is the lever which controls it in its backward or forward movements. When the head is in line with the neck and body, it is controlled by power acting upon the jaw, at right angles to the neck. When the head is thrown back, so as be at an angle to the neck, the muscular power is applied to the jaw, more in a line with the neck, or in certain modifications of it. In other words with the head, neck and body upright, the muscles were supposed to act upon the head vertically, or in modifications of that direction ; when, in fact, they act horizontally ; or the chin was pulled down to flex the head, when in fact it is pulled back. In throwing the head back out of balance, the muscles in the back of the neck draw down vertically, while the digastric relaxes and allows the chin to go outward and upward. To bring the head forward, the genio-hyoid comes into action to make up for the loss of power in the digastric, which is then acting less directly and has also to contend with a larger portion or weight of the head, behind the atlas ; as the head comes into balance, the genio-hyoid forms a more decided angle with the neck, by which it loses its vertical power and ceases to act. During these movements, there is no sudden cessation of the action of any muscle, but those which act vertically come into action, as those which act horizonitally relax ; and the reverse. But in sudden movements of the body forward, or in abrupt checks to its backward motion, the genio-hyoid acts instaneously to start the head, in the first instance, and to check it in the last ; its service in these movements being modifications of that rendered by it in rising from the back. Thus the head is always under control. If, however, the muscles acted vertically, or in modifications of that direction as hitherto supposed, the head could not be controlled steadily, for the digastric would have little power and the genio-hyoid none whatever, until the head had gone up considerably, and had acquired such momeutum as to be unmanageable. If, therefore, the omo-hyoid muscle acted in common with those which pass up the front of the neck, and the other muscles which centre in the hyoid bone, as hitherto supposed, locomotion would be destructive, the head would fall back upon any sudden movement forward, and also in the attempt to rise from the back. If the posterior fibres of the mylo-hyoid, with the hyo-glossus and muscles above, attempted to preserve the balance, the hyoid bone would be torn apart, unless the styloid processes and the palate muscles, etc., gave way, as the weight of the head would be uncontrollable by these muscles, because of their want of leverage, whereas, the head, as now shown, is balanced easily and controlled effectually, by muscles acting upon a long lever, in every position of the head, neck and body. The action of the parts, under the loss of even all the jaw, presents nothing to disprove these conclusions, as the omo-hyoid, the digastric and other muscles, still act, while those which would be injured attempt nothing inconsistent with their structure and position. The effect of the loss of the jaw upon the surrounding parts (as far as my observation extends) proves that the action of the muscles is mostly horizontal, for the chin is drawn back very decidedly and the face *shortened.* Notwithstanding this, in opening the mouth (which can be done only to a small extent) the movement still seems to be backward, as much as downward, even when the substitute for the jaw is hinged in the glenoid fossæ, and does not come forward like the condyles of the jaw.

In giving all due credit to these muscles which act upon the head horizontally, it should be borne in mind that those of the neck which act vertically, exert important influence; for, without their fixation of the hyoid bone, the digastric muscle could not move the head, as it would, when acting, draw in a straight line between the digastric notch and jaw, and with the latter fixed, it is virtually part of the head. With the hyoid bone held firm however, the posterior belly of the digastric slants down, while the anterior belly goes horizontally forward to the jaw. In this way a slight contraction, of both portions of the muscle has a very important effect upon the head, as there is enough movement of the tendon, through the loop, to allow the contraction of both bellies of the muscle to bear upon the jaw, and thus draw in the chin sufficiently to keep the forehead forward, without pulling the hyoid bone far back. If the digastric acted vertically however, (even throwing out the effect supposed to be exerted by both bellies in drawing the head backward) only half, the strength of the muscle would affect the head, to bring it down in front, (as the muscle would be acting from the hyoid bone) the other half being exerted against the digastric notch, the origin of the posterior belly. There would also be the disadvantage of requiring much more contraction in the muscle, and, of its action being less direct, even allowing, that the hyoid bone could descend ; this, however, is impossible, and the action of the digastric is but little approximated to the vertical direction, even when the head is thrown up. It is probable that the stylo-hyoid muscles (the ligaments certainly do) assist the muscles of the neck in holding the hyoid-bone back, particularly during strong action of the genio-hyoid, as this would give greater freedom to the digastric muscle.

Thus the powerful muscles which are attached to the occipital bone, behind the condyles, and act upon the head vertically, are antagonized by muscles which act upon the jaw, principally in a horizontal direction, and the head is effectually controlled, notwithstanding the little power of the muscles attached to the occipital bone in front of the condyles.

The following quotations show the opinions entertained as to the action of the muscles which move the lower jaw:

Gray's *Anatomy* says : "The temporal, masseter and internal pterygoid raise the lower jaw against the upper with great force. The two latter muscles, from the obliquity in the direction of their fibres, assist the external pterygoid in drawing the lower jaw forward upon the upper, the jaw being drawn back again by the deep fibres of the masseter, and posterior fibres of the temporal. The external pterygoid muscles are the direct agents in the trituration of the food, drawing the lower jaw directly forward, so as to make the lower jaw project beyond the upper. If the muscle of one side acts, the corresponding side of the jaw is drawn forward, and the other condyle remaining fixed, the symphysis deviates to the opposite side. The alternation of these movements on the two sides produces trituration.''*

Todd & Bowman's *Physiological Anatomy* part iii., p. 539, says: "The *external pterygoid* neither raises nor depresses the lower jaw.''

My own views as to these muscles also, differ materially on some points from those expressed in the quotations. The *stylo hyoid ligaments* make it impossible that the hyoid system of muscles can depress the lower jaw by acting upon it vertically, and the following experiments show that they do not.

By resting the finger on the thyroid cartilage, with its end placed against the hyoid bone, it will be found that they do not descend when the jaw is opened.† Further, if the thumb is placed under the chin and the jaw held *firmly* open against it, by carefully throwing the hyoid bone up by swallowing, it will be found that the jaw is held down by other muscles than those inserted into the hyoid bone; as it will not go up if the experiment is properly conducted, even when the hyoid bone is carried above the border of the jaw. This may, however, require some care and practice; as the jaw is, in general, nearly shut in deglutition, and its depressors are inclined to relax in sympathy with the movements of the other parts. The position of the *masseter* is too well known to need particular description. The fibres of the deep portion have a more perpendicular direction than those of the superficial

---

*Gray's Anatomy, Descriptive and Surgical. 2d American Edition, p. 252. Phila., 1865.

†Except to a trifling extent, when the jaw is opened unusually quick or wide.

portion, the last passing backward as much as downward. The deep portion draws the jaw upward and backward, the superficial portion upward and forward. The *internal pterygoid* has the same general direction as the superficial portion of the *masseter*, excepting that as it arises from the pterygoid fossa it passes considerably outward to reach its insertion on the inner side of the ramus and angle of the jaw. It has, therefore, not only the upward and forward motion of the superficial portion of the masseter, but also lateral power over the jaw.

These muscles not only raise the jaw and teeth, when cutting with the incisors and crushing with the molars, but are also the main movers of the jaw in trituration. In cutting they bring the jaw forward bodily ; in triturating they exert more forward and lateral action on one side than on the other ; by this the jaw is thrown over to the opposite side and then *drawn in and carried out continuously on that side*, and not carried over to the other side. It is a mistake to suppose that trituration of the food is effected by the alternate action of the muscles of both sides. When the jaw and teeth are perfect, the teeth of one side only are used, until the muscles tire, perhaps, and then those of the other side are resorted to. If the teeth are tender, badly placed, or deficient on one side, trituration is performed on the other only.

The *temporal* muscle, which covers so large a portion of the side of the head, and is strongly inserted into the inner surface, apex and anterior border of the coronoid process of the jaw, pulls its insertion upward and backward, and assists the *masseter* and *internal pterygoid* muscle in cutting, crushing and triturating the food.

The *external pterygoid* is a short, thick muscle, which arises from the pterygoid ridge on the great wing of the sphenoid, and the portion of bone included between it and the base of the pterygoid process, from the outer surface of the external pterygoid plate and the tuberosity of the palate and superior maxillary bones. It arises in two portions separated by a short interval; they both pass outward and backward and are inserted into a depression in front of the condyle of the lower jaw, and into the corresponding part of the interarticular fibro-cartilage. The separate portions join and form one muscle previous to their insertion; the middle fibres being horizontal, but as the origin of the muscle is very wide vertically, the upper portion descends in passing back, while the lower ascends. This strong and beautiful muscle has, from its peculiar situation, great influence over the jaw. The origin being more internal than the insertion, gives the muscle control over the condyle laterally, by which it is held firmly in the glenoid cavity, and the great strength of the muscle tends to keep the condyle from being driven back by falls, or blows upon the chin, which otherwise might easily occur, as the glenoid fossa is very shallow behind.

A more prominent service of this muscle is when it brings the condyle forward either on its own side alone, as in trituration, or with its fellow of the opposite side, as in cutting by the incisors. In these movements it acts in concert with other motors of the jaw. To consider it especially the triturating muscle is no more correct than to suppose that when triturating with a pestle in a mortar, the thumb and forefinger (which are further from the grinding surface) are the triturators rather than the fingers below.

The *external pterygoid* muscle controls the upper end of the jaw in concert with the *temporal*, while the muscles attached to the body have especial control below, and by the concerted action of all, power and steadiness are secured in the complicated movements of the jaw, and of the lower teeth against the upper. The *external pterygoid* muscle has one office peculiar to itself, that of holding the condyle fixed on any part of the eminentia articularis, to which it may be drawn in movements of the jaw. For this service the muscle is admirably fitted by the great width of its origin, which enables it to brace the condyle so firmly against the part above and in front of it, that the jaw is fixed, even when wide open, as firmly as if the condyle were hinged in that position.

The *eminentia articularis,* the rounded projection which forms the front of the glenoid fossa, is indispensable to this fixation of the condyle; and as the condyle in coming forward mounts this eminence, the jaw, while going down in front, is also carried down bodily, by which the back teeth of the lower and upper jaws are widely separated. These advantages are gained while the centre, or rather the curve, upon which the jaw turns, is three-fifths down toward the angle. This may be tested by placing the wrist and hand around the back of the neck and applying the end of the middle finger to the back of the ramus, and finding the point upon which it turns, which is probably near the insertion of the internal lateral ligament. By this arrangement the jaw is opened promptly and widely, without interfering with the parts behind; which would be injuriously pressed upon if the condyle remained still and the angle went back far enough to open the jaw wide.

The *external pterygoid* is now to be spoken of in a service in which the action of the muscle is greater in range, frequency and importance than in any other, and in which it has never before been recognized, *that of opening the mouth.* This muscle antagonises the temporal, masseter and internal pterygoid muscles and is, especially by its lower head, *the depressor of the lower jaw.* It does this alone when necessary, without assistance from any other muscle. Its ability to do this may be proved by bracing the arm against the breast and applying the thumb firmly to the chin; or better, by placing the jaw flat on the mantle, or any fixed support, then on opening the mouth the head will go backward, being drawn back by the external pterygoids whose insertions in the condyles are fixed by support of the jaw.

The action of the external pterygoid muscles in opening the jaw is similar to their action when the jaw is brought forward to cut with the incisors, the difference in effect being produced by other muscles ; the temporals probably act similarly in both movements. The masseters and internal pterygoids, however, must relax in the opening of the jaw instead of assisting to carry it forward as in cutting, while the digastrics, which relax in the forward movement of the jaw, undoubtedly assist to draw the chin back in gaping, vomiting, and in very wide opening of the mouth when voluntary. When not otherwise employed, it is probable that they always assist in opening the jaw, as they are admirably fitted for carrying the chin back, when the condyle is going forward, It is impossible that they could carry it back readily if the condyles were not at the same time pulled forward. For, when the chin points down very much (as in some persons even with the jaw closed), the digastric muscle forms but a slight angle at the loop, and decided action of it in such cases would tend to keep the condyle of the jaw back in the glenoid cavity from the start, or soon after, as the opening of the jaw would straighten the digastric so much that it would pull nearly in the direction of the ear and have but little power to carry the chin back. That the digastrics draw the condyles of the jaw back in the glenoid fossæ when acting without decided action of the external pterygoids, is demonstrated by exerting strong suction with the tongue against the roof the mouth. In doing this the upper and lower teeth are held a little apart and the condyle of the jaw can be felt to move back, as the muscles tighten, if the point of the little finger is pressed firmly into the ear so that its front rests against the auditory process. In cases where the anterior belly of the digastric ascends from its loop to the chin, so that it has some vertical direction to favor it; and even supposing it were assisted by strong back fibres in the mylo-hyoid muscle, it is not possible that the condyle could release itself from the *eminentia articularis ;* and unless it did so and came forward, the jaw could open only to a trifling extent, for it would act as if hinged in the glenoid fossa. But with the external pterygoids drawing the condyles forward, the jaw opens readily, as upon a centre in the ramus, and the question arises as to what the jaw really turns upon.

The masseter and internal pterygoid muscles hold the angle very much as if a sling were passed around it, while the *stylo maxillary ligament* is also inserted into the angle. It might therefore be thought that the jaw turned upon the angle if this part did not go back during the forward movement of the condyle, thus proving that the jaw turns on a portion of the ramus between the angle and the condyle. Under all the circumstances the insertion of the *internal lateral ligament* around the inferior margin of the dental foramen marks the centre upon which the jaw opens with sufficient exactness. This ligament is a thin aponeurotic expansion, which descends perpendicularly from the extremity of the spinous process of the sphenoid bone, and cannot properly be considered the special support of the jaw in its downward or upward movement. It is more likely that the pterygo-maxillary ligament also exerts an influence, and that all the ligaments and muscles inserted into this part of the bone are instrumental in determining the centre upon which the jaw opens.

The shape of the jaw, whether congenital or modified by age, or accident, may also vary the location of the centre somewhat; but in every case the condyles must come forward to open the jaw effectually. This can be done only by the *external pterygoid* muscles, whose special office is to move and also fix the condyles, and in connection with the temporal and other elevators, the jaw also. In this way only can the digastrics and associate muscles act efficiently in their most important functions; otherwise they would be disturbed by undue movement of the jaw. Sometimes, however, the digastrics, as before stated, assist in moving and holding the jaw.

When the *external pterygoids* throw the jaw wide open, the chin is much below the hyoid bone, but controlled by the anterior bellies of the digastric muscles, the hyoid bone being fixed by the posterior bellies and by the *omo-hyoid* muscles, which can now be felt in action. Consequently the three extremities of the jaw, the chin, the angle, and the condyle, have each diverging or converging muscular support. The *digastrics* go to the chin, the *internal pterygoids* go to the angles, and the *external pterygoids* to the condyles. This arrangement of the muscles in connection with the ligaments holds the jaw firm in all its complicated movements.

It has been shown that the *sterno-cleido-mastoid* muscles do not rock the head, but that they give anterior support to the atlas, while the splenii muscles give posterior support, and that in combination they support it and the head laterally. This support is so complete that the head has a firm resting place, upon which it is held securely in all positions. The muscles which give form to the neck, and support and move the spine and head, are so arranged as to leave the front of the spine free for the tongue, larynx, and trachea, with their special muscles, &c., and for nerves, blood-vessels, and glands of prime necessity.

These important parts are shut in and protected by the lower jaw, which gives attachment to muscles indispensible to those organs in the performance of their functions, while its own movements are in strict subordination to them. By this respiration is secured from interruption at all times, and occasionally made tributary to vocalization and articulation, and the vocal tube modified and moved with wonderful delicacy and energy in its participation in these functions, and in accordance with those of mastication and deglutition.

The lower jaw is also the great lever by which the head is held upright.

These explanations of the physiological action of the muscles which control and influence the lower jaw, prepare the way for the diagnosis of its fractures, upon which I purpose to write hereafter.

